# Inflammasome activation as a link between obesity and thyroid disorders: Implications for an integrated clinical management

**DOI:** 10.3389/fendo.2022.959276

**Published:** 2022-08-19

**Authors:** Rosario Le Moli, Veronica Vella, Dario Tumino, Tommaso Piticchio, Adriano Naselli, Antonino Belfiore, Francesco Frasca

**Affiliations:** Endocrinology Unit, Department of Clinical and Experimental Medicine, University of Catania, Garibaldi-Nesima Hospital, Catania, Italy

**Keywords:** obesity, inflammasome, thyroid autoimmunity, weight regain, thyroid cancer, Lt-4 therapy

## Abstract

Obesity is strongly associated with chronic low-grade inflammation. Obese patients have an increased risk to develop thyroid autoimmunity and to became hypothyroid, suggesting a pathogenetic link between obesity, inflammation and autoimmunity. Moreover, type 2 diabetes and dyslipidemia, also characterized by low-grade inflammation, were recently associated with more aggressive forms of Graves’ ophthalmopathy. The association between obesity and autoimmune thyroid disorders may also go in the opposite direction, as treating autoimmune hyper and hypothyroidism can lead to weight gain. In addition, restoration of euthyroidism by L-T4 replacement therapy is more challenging in obese athyreotic patients, as it is difficult to maintain thyrotropin stimulation hormone (TSH) values within the normal range. Intriguingly, pro-inflammatory cytokines decrease in obese patients after bariatric surgery along with TSH levels. Moreover, the risk of thyroid cancer is increased in patients with thyroid autoimmune disorders, and is also related to the degree of obesity and inflammation. Molecular studies have shown a relationship between the low-grade inflammation of obesity and the activity of intracellular multiprotein complexes typical of immune cells (inflammasomes). We will now highlight some clinical implications of inflammasome activation in the relationship between obesity and thyroid disease.

## Introduction

**Figure 1:**
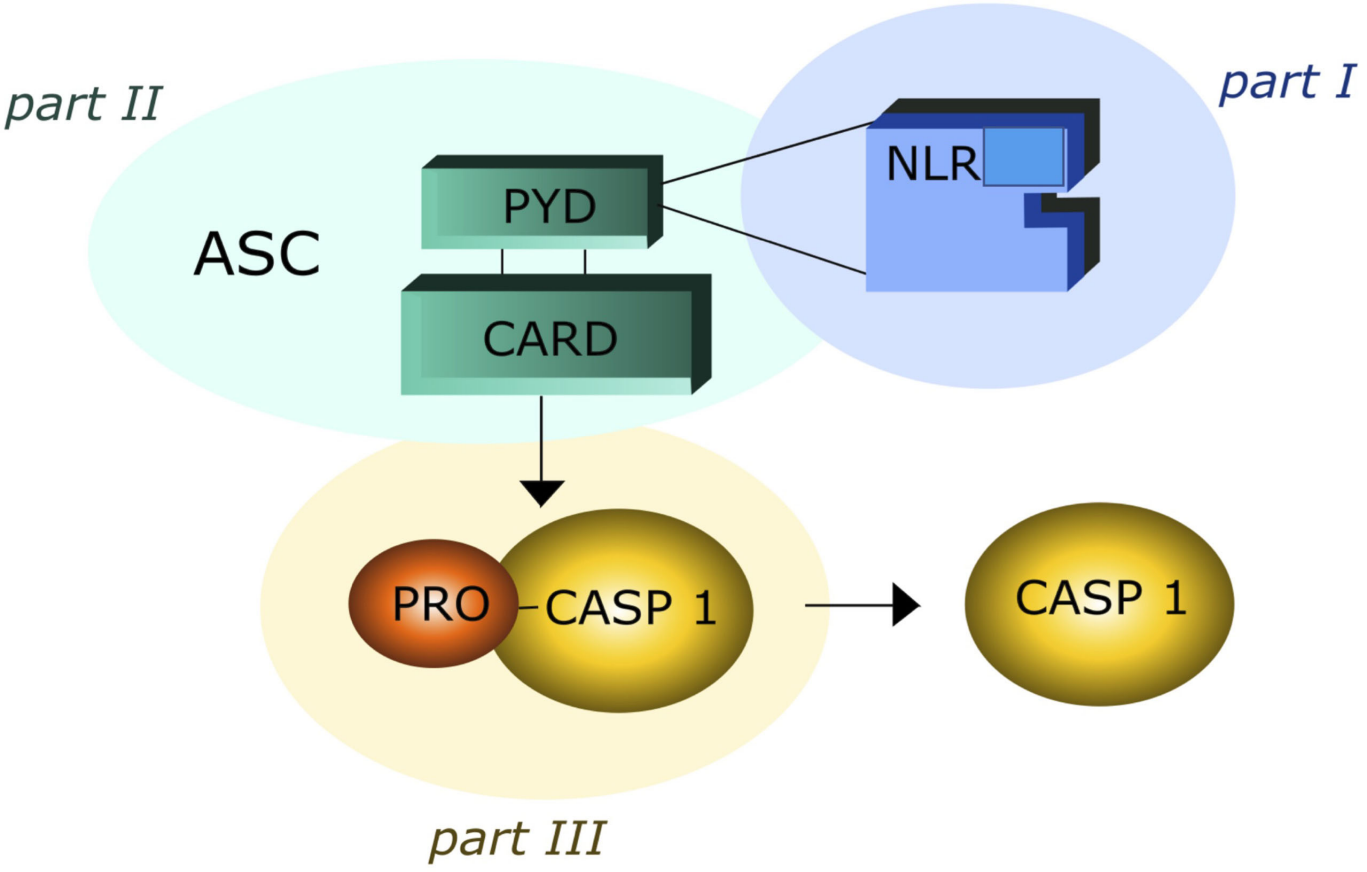
The three elements of inflammsome oligomeric structure: part I contain Nod-Like Receptor (NLR); part II contain Adaptor Protein Apoptosis-Associated Speck-Like Protein (ASC), Piriin Domain (PYD), Caspase Recruitment Domain (CARD); part III contain Pro Caspase I (PRO –CASP-1).

Obesity is a public health concern with a prevalence rapidly increasing in the last ten years from 3.2% to 10.8% in adult men and from 6.4% to 14.9% in adult women (1). Adipose tissue should be considered an endocrine organ comprising various cell types such as adipocytes, preadipocytes and immune cells (2). Adipocytes are prone to secrete pro-inflammatory adipokines, such as tumor necrosis factor-α (TNF-α), monocyte chemoattractant protein-1 (MCP-1), and interleukin-6 (IL-6), which recruit immune cells and amplify the inflammation process (3, 4). These mechanisms may play a major role in obesity-related disorders, including atherosclerosis, diabetes, neurodegenerative diseases and cancer (5–7). New emerging data reveal a relationship between the low-grade inflammatory state of obesity and inflammasome activation level (8, 9). Inflammasomes are intracellular multiprotein complexes typical of immune cells, such as monocytes and macrophages, which mediate the first line of defense in response to both sterile (absence of microbial and virus particles) and non-sterile (microbial and/or virus infection) injuries, leading to the processing and release of pro-inflammatory cytokines (10–16). The inflammasome oligomeric structure includes three elements: I) NOD-like receptor (NLR), that is a stress signal receptor (17–19). NLRs can contain a Pirin Domain 3 (NLRPD3) or a caspase recruitment domain (NLRC4) and are present in several cells of different tissues; II) adaptor protein, that is an apoptosis associated speck-like protein containing caspase activation recruitment domain (ASC); III) pro-caspase-1(pro-CASP1), pro-IL-β and pro-IL-18 (**Figure 1**).

A further mechanism that contributes to the background activation of inflammasome in obese patients is the reduced efficiency of innate immunity caused by the inhibition of autophagic processes (20, 21). This mechanism contributes to the activation of the NLRPD3 inflammasome, which in turn (22) enhances chronic low-grade inflammation in adipose tissue (23, 24), thereby resulting in systemic insulin resistance and metabolic impairment (25). Here we highlight some emerging clinically relevant implications of inflammasome activation and chronic low-grade inflammation associated with obesity for patients with thyroid disorders.

## Obesity, inflammasome and immune related thyroid diseases

**Figure 2:**
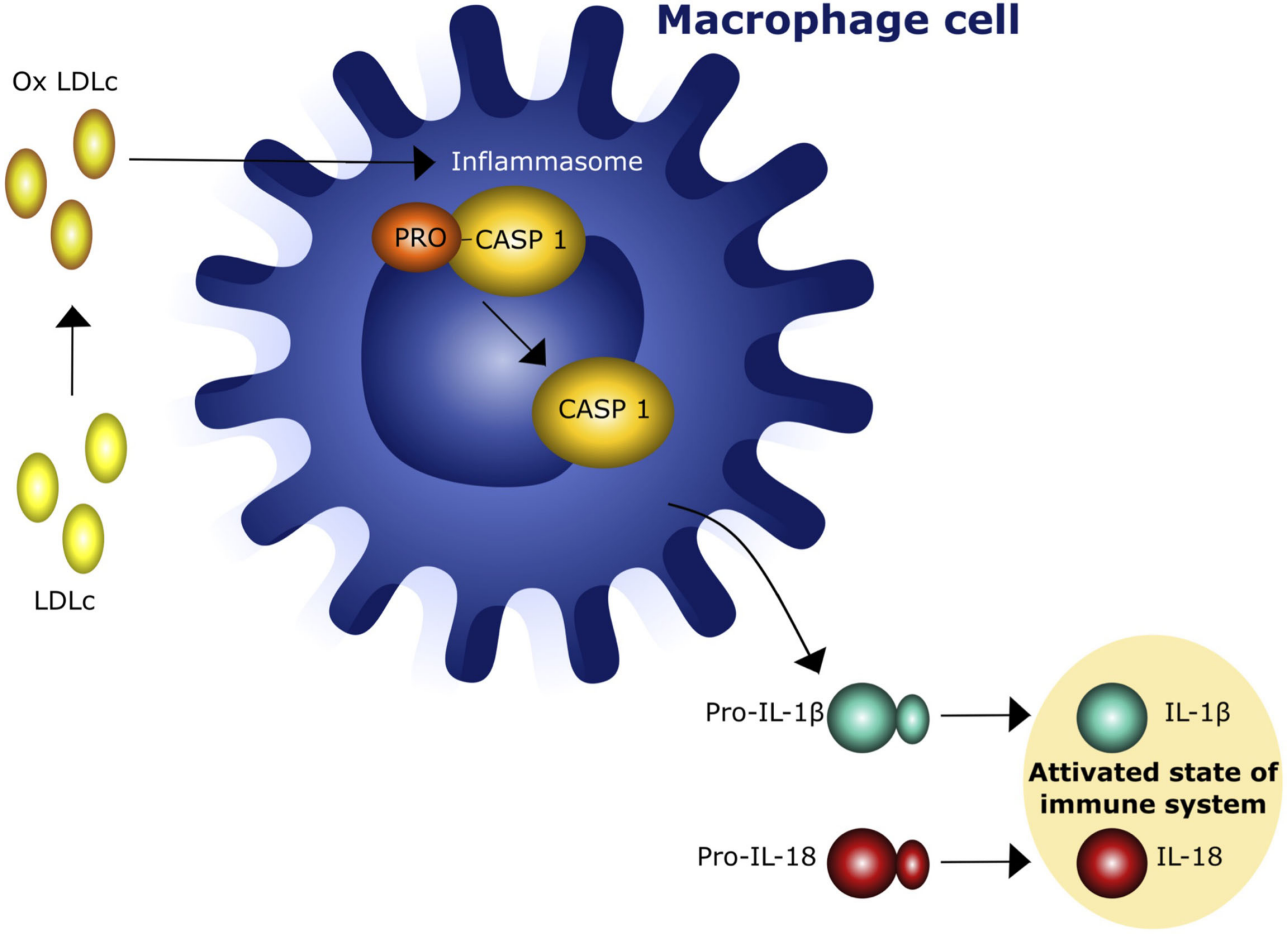
Interaction of oxidized low density lipoprotein cholesterol (oxLDLc) with cells of innate immunity. IL - 1β = Interleukin 1β, IL-15 = interleukin 18.

**Figure 3:**
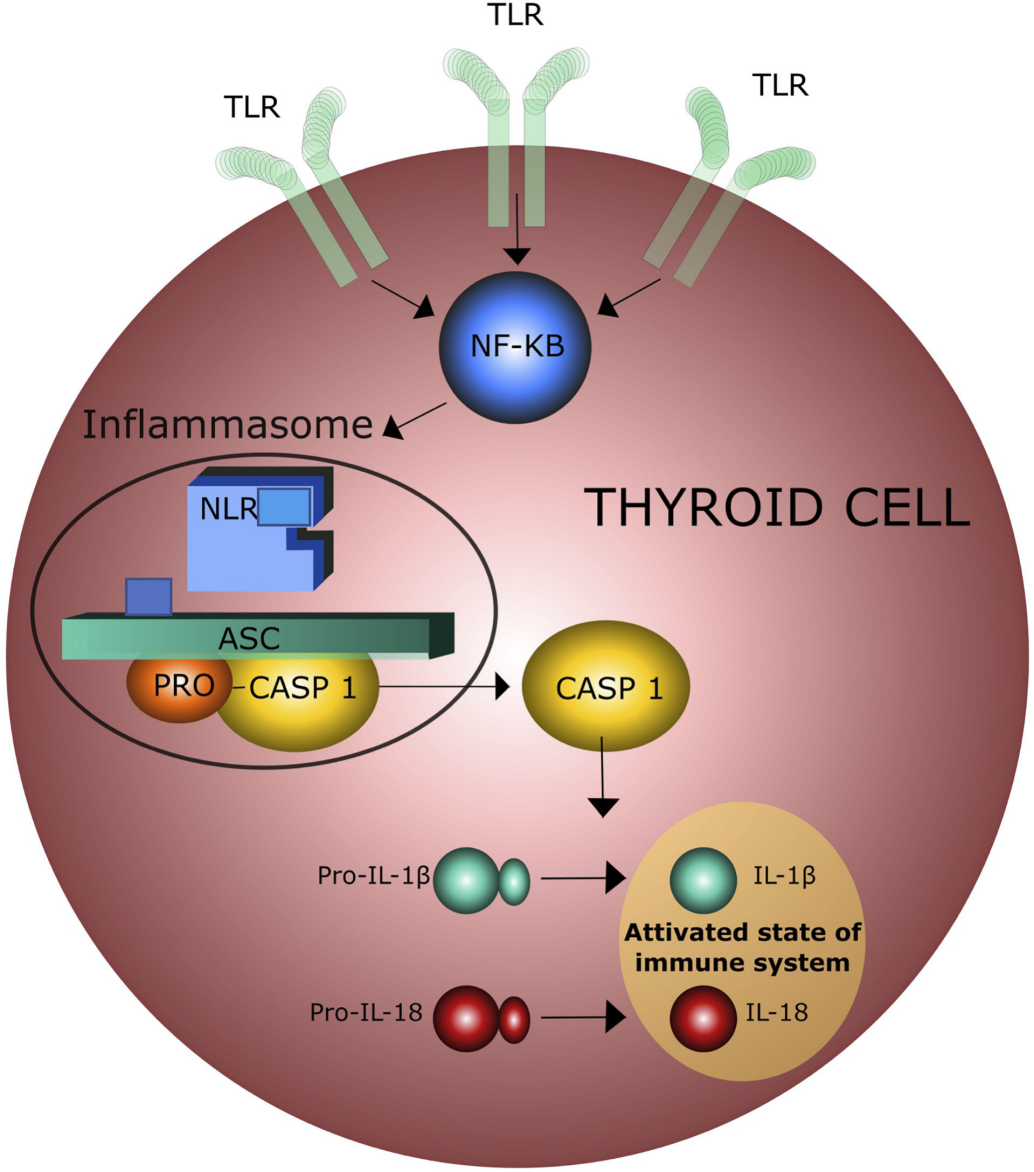
Activation of inflammasome by toll-like receptors (TLR) in thyroid cells; NF-KB = Nuclear Factor – KB.

Recent epidemiological and experimental data provided evidence of a bidirectional interaction between obesity and thyroid autoimmunity (26–28). In particular, obesity is associated with an enhanced risk of hypothyroidism and an increased risk of developing anti-thyroperoxidase antibodies (TPOAbs) (27). One prospective study of 783 obese patients referred to bariatric surgery reported a prevalence of 17.1% and 12.3% of autoimmune thyroiditis and autoimmune hypothyroidism, respectively (29). This association was confirmed by the National Health and Nutrition Examination Survey (NHANES III) which described the presence of TPOAbs in 11.3% and hypothyroidism in 4.6% of 17,353 patients (30). Autoimmune thyroid disease (AITD), including Graves’ disease (GD) and Hashimoto’s disease (HT), include thyroid disorders characterized by an immune reaction against thyroid autoantigens that occur in individuals with a distinct genetic background after exposure to certain environmental factors (31–36). GD and HT share similar pathological features characterized by lymphocyte infiltration of the thyroid tissue and antibodies production (37). The relationship between obesity and thyroid autoimmunity is not only based on epidemiological aspects but also on common pathogenetic mechanisms. A major player of this association is insulin resistance (IR), that is present in 15.5 - 46% of the obese patients (38). IR attenuates the insulin-stimulated phosphoinositide 3-kinase (PI3K) signaling pathway, causing hyperinsulinemia and a variety of adverse effects, including increased oxidative stress due to the production of reactive oxygen radical species (ROS) which activate the inflammasome complex in different tissues (25, 39) and reduces the vasoactive/anti-inflammatory effect of nitric oxide (NO) (39, 40). A variable degree of leptin resistance (LR) is usually associated with IR and obesity and can be considered a second mechanism in the interplay between obesity and thyroid disorders. Indeed, chronic exposure of endothelial cells to increased circulating levels of leptin also leads to a decrease in NO production (41). In addition, ROS induce pro-inflammatory MCP-1 expression which further enhances leukocyte infiltration into vascular cells. Chronic activation of inflammatory response contributes to the impairment of a correct immune response to exogenous antigens and to the persistence of autoimmune processes in predisposed subjects (42–44). This concept is supported by the observation that increased NLR inflammasome activity in both obese patients and experimental animals can play a major role in macrophage recruitment and immune activation (45). The third mechanism is the increased oxidation of low-density lipoproteins (LDL) occurring in obese patients with IR. Several lines of evidence indicate that increased oxidized low-density lipoproteins (oxLDLc) and cholesterol crystals can trigger the activation of NLR inflammasome, that, in turn, leads to a tonic activation of innate immune system (45, 46) (**Figure 2**). Hence, insulin/leptin system dysregulation along with oxLDLc increase and inflammasome activation are associated with impaired immunological tolerance and metabolic changes favoring thyroid autoimmunity (41, 45–48). The complete NLR-mediated inflammasome activation in thyroid cells is a complex event that involves the engagement of the tool - like receptors (TLRs) and the recruitment of the myddosome complex, which triggers downstream nuclear factor - KB (NF-kB). cell signaling, resulting in the increased levels of NLR and pro-ILs (49, 50) (**Figure 3**).

**Figure 4:**
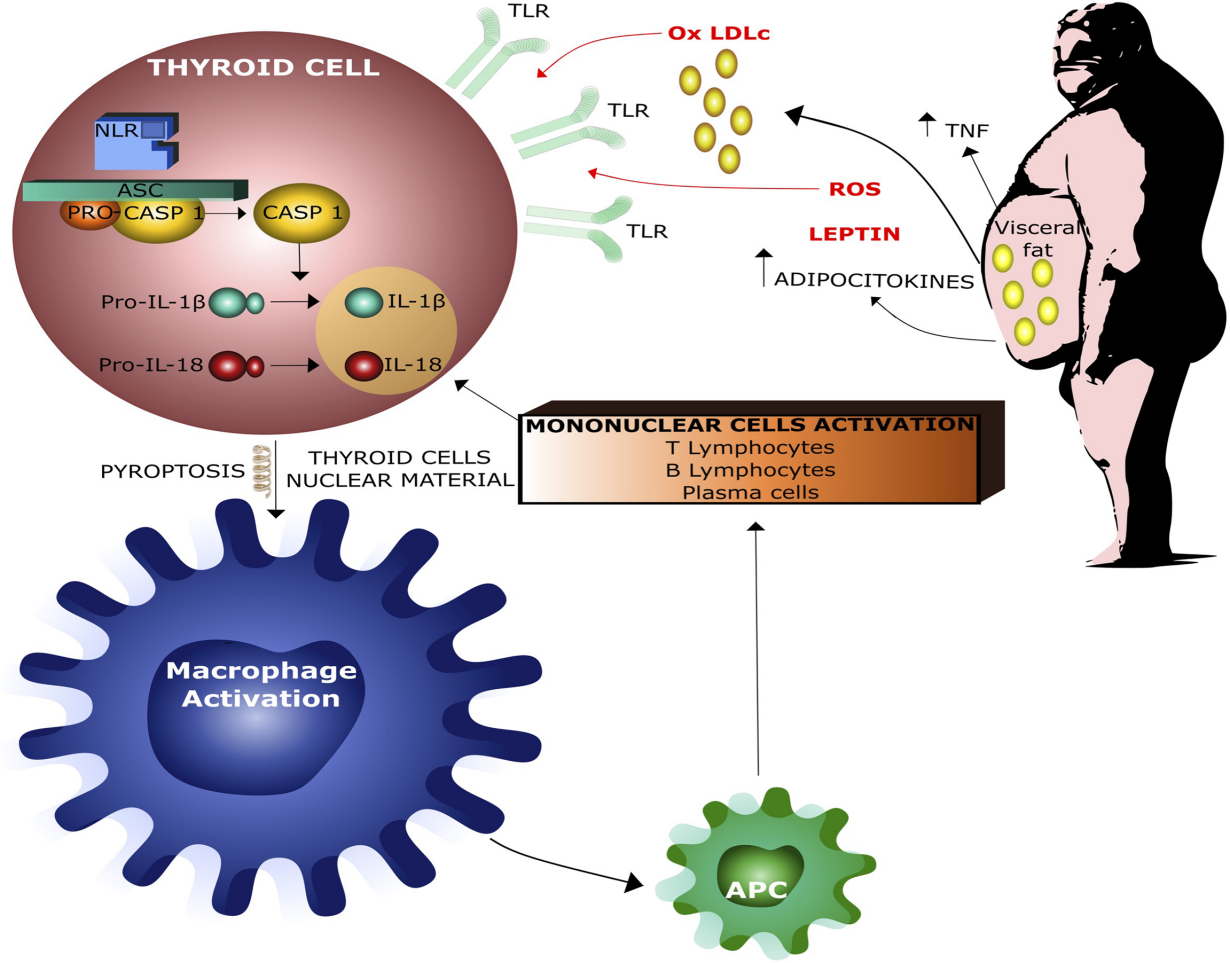
Interaction between obesity and thyroid damage by infammasome activation. Ox LDLc = Oxidized Low Density Lipoproteins Cholesterol, ROS = Radical Oxigen Species, APC = Antigen Presenting Cells.

More specifically, low-grade inflammation causes a predominance of CD4 + T helper cells over the proinflammatory Th17 subsets, increasing the IL-1β production which plays a major role in the pathogenesis of Hashimoto’s thyroiditis (HT) (40, 51, 52). It is interesting to note that both human and rat thyroid cells express functional TLRs on their surface and these receptors may directly bind both endogenous and exogenous ‘danger molecules’, thereby promptly activating the innate immune system. Several experimental evidences support the idea that the innate immune response is linked to thyroid tissue damage. Thyroid cell damage leads to the release of intracellular proteins that can be recognized by antigen-presenting cells (APCc), thereby activating acquired immune response, which, in turn, amplifies thyroid cell damage and ultimately causes thyroid tissue destruction (35, 53–55) (**Figure 4**).

Thyroid hormone are another important component of the putative bidirectional cross-talk between obesity and thyroid immunity process. All clinical conditions causing altered thyroid hormone levels may contribute to the extent of activation of inflammation and immunity response. On the other hand, alterations in thyroid hormone levels may favor obesity, atherosclerosis, and inflammation. Recent studies indicate that triiodothyronine (T3) negatively modulates NLR inflammasome and macrophage function. Conversely, low levels of thyroxine (T4) may contribute to inflammation and immune response activation in hypothyroid patients (56–59). This effect of T4 may also occur through an extra-genomic pathway by binding the cell membrane integrin receptor avβ3, which, in turn, may modulate the immune response *via* the activation of signaling pathways including mitogen-activated protein kinase (MAPK), cyclooxygenase 2 (COX-2) and hypoxia factor 1 alpha (HIF-1a), all converging into inflammasome activation (60). Taken together, these studies suggest that thyroid hormone effect on immune response is indirect and can be mediated by the combination of both genomic and nongenomic actions in specific cellular/clinical contexts (61) and these mechanisms are of primary importance in obese patients who frequently display reduced T4 levels (56).

### Key points

Available evidence suggests a two-way interaction between obesity and inflammation and autoimmunity. In obese, insulin-resistant patients, careful attention to thyroid hormone homeostasis can prevent worsening of metabolic conditions and prevent thyroid dysfunction.

## Obesity, inflammasome and thyroid eyes disease

Graves’ ophthalmopathy (GO) is an autoimmune thyroid related eyes disease (TED) occurring in about 25% of patients with Graves’ disease (GD). Elevated levels of thyroid stimulating receptor antibodies (TRAbs) are considered a risk factor for severe GO. Gender, age, smoking, and duration of hyperthyroidism are additional risk factors for the severity of GO (62–64). Recently, diabetes mellitus (DM) has been related to GO severity. Moreover, the prevalence of Type I diabetes is higher in patients with GO than in the normal population and dysthyroid optic neuropathy (DON) occurs more frequently in patients with both GO and DM and have a worse prognosis than in those with GO alone (65). Moreover, one important study showed evidence that DM and cigarette smoking are significant predictors of severe GO. In particular, DM was a stronger determinant of GO severity (p=0.001) and significant predictor of diplopia (p= 0.03). Additionally, in Graves’ patients with Type II diabetes (T2DM), GO preceded the onset of hyperthyroidism more frequently than in control patients (p < 0.05). GO severity was significantly associated to T2DM with a duration longer than five years (Odds Ratio = 4.9; p = 0.045) and the presence of micro and macro vascular complications (Odds Ratio = 4.8; p = 0.048). GO severity was also related to patient overweight (BMI > 26) (66). These clinical evidences suggest that GO may be related to a peculiar pathophysiologic background linked to the metabolic syndrome and obesity. Indeed, insulin-resistance and compensatory hyperinsulinemia in obese patients may exert proliferative effects on human orbital preadipocytes/fibroblasts *via* the phosphatidylinositol 3-kinase (PI3-K) and the mammalian target of rapamycin (mTOR) signaling pathways (67). In addition, elevated levels of insulin increase IGF-1 bioavailability, induce overexpression of the IGF-I receptors (IGF-IR) in orbital preadipocytes/fibroblasts and enhance IGF-IR/TSHR cross-talk thereby contributing to the severity of GO (68–72). Systemic low-grade chronic inflammation causes NLR inflammasome activation in fat tissues and production of neurotoxic factors such as IL-1β and TNF-α, that are determinants of GO pathogenesis (73). Moreover, low-density lipoproteins cholesterol (LDLc) and their oxidized forms are increased in obese and diabetic patients leading to reduced or incomplete autophagic processes (74) and to the activation of NLR inflammasome in macrophage cells. In GO of obese patients, activated macrophage population within the orbital microenvironment may increase the secretion of IGF-1 by fibroblasts thereby promoting adipogenesis, hyaluronan synthesis and the expansion of soft orbital tissues (50, 75). Stein et al. reported that statins are able to reduce the onset of GO in patients with GD. A recent cross-sectional study indicated that total and LDL cholesterol is associated with GO activity, suggesting that high serum cholesterol level is a risk factor for GO development (64, 76). Considering that GO is an inflammatory and autoimmune response against thyroid and orbital autoantigens, statins may protect from GO presentation and activity owing to their anti-inflammatory and hypolipemic action. Hypercholesterolemia can cause chronic inflammation per se through oxLDL by modulating inflammasome activity *via* TLRs and increasing the expression of dipeptidyl dipeptidase IV (DPP4) in macrophages. Raised DPP4 activity leads to an increase of CD36+ cells, which is a hallmark of the atherosclerotic inflammatory process in obese and insulin resistant patients (50). In particular, LDLc may attenuate corticosteroid transcriptional and translational activities thereby reducing their anti-inflammatory efficacy in GO (77). Taken together, these observations support the notion that chronic low-grade inflammation and inflammasome activation in dysmetabolic subjects may sustain the autoimmune process underlying TED pathogenesis and their clinical and phenotypic expression.

### Key points

Obesity, diabetes and hypercholesterolemia contribute to GO disease. The use of statins and strict cholesterol control should be considered as part of the GO treatment plan.

## Therapy of hyperthyroidism, cytokines, and weight gain

It is a common observation that hyperthyroidism is associated with weight loss in most cases, while approximately 10% of hyperthyroid patients experience weight increase. Therefore, restoration of euthyroidism usually results into weight regain that is sometimes excessive. The mechanism underlying this phenomenon is not entirely clear and it is commonly believed that the transition from hyper to euthyroidism can promote obesity in predisposed patients (78). On the other hand, some studies tried to identify specific risk factors for such weight increase that may include the severity of the autoimmune process in Graves’ disease (78, 79). Data regarding post-therapy body composition (lean and fat body mass) in terms of preferential accumulation and timing are still conflicting (79, 80). However, recent studies focus on the effect of dietary programs in the prevention of weight gain compared to standard care (81). Taken together, these studies strongly suggest that weight regain after treatment of hyperthyroidism is not only caused by a decreased catabolic effect of thyrotoxicosis, but rather linked to additional, independent mechanisms (82). This hypothesis is supported by the observation of a relationship between treatment modalities for hyperthyroidism and weight regain, with a greater effect for radioiodine and surgery, compared to anti-thyroid drugs. Indeed, several studies indicate a statistically and clinically significant excess of weight gain after thyroidectomy for hyperthyroidism (83). However, this observation is mitigated by studies showing body weight gain in all thyroidectomized patients regardless of underlying thyroid disorder (83, 84). Accordingly, excessive body weight gain after thyroidectomy has been attributed to deficient replacement therapy with L-T4 and to an imbalance of thyroid hormone homeostasis due to poor tissue conversion of T4 to T3 (85). However, several studies indicate that radioiodine (RAI) therapy is also associated with significant weight gain (by 5-6 kg on average over 1year post-treatment). This weight gain can last up to 5 years and lead to excess body weight compared to the premorbid condition similarly to what is observed in Graves’ patients after total thyroidectomy. Treatment of hyperthyroidism with anti-thyroid drugs (ATDs) is also associated with 2-4 kg weight gain which starts after the start of the initial treatment and may last one year after the end of the treatment (86, 87). This effect occurs with both treatment with ATDs alone and block-and-replace therapy regimen (ATDs plus L-T4) although studies directly comparing these two regimens are lacking (88). However, there is no clear evidence that ATD treatment leads to lasting weight gain. Conversely, several observations indicate that body weight remains unchanged after completion of ATD therapy, suggesting that the impact of these drugs on weight and energy balance differs from surgery and radioactive iodine and depends on residual thyroid function (84). These considerations were reinforced by a comparative study between different treatment modalities for hyperthyroidism. Indeed, a retrospective study including 133 patients with Graves’ disease who received ATDs, surgery or RAI indicated that treatment of hypothyroidism post-thyroidectomy or post-RAI was associated with significantly greater weight gain compared to patients who achieved euthyroidism with ATDs or hemi-thyroidectomy (78). Interestingly, this study found that these Graves’ patients experienced more weight gain compared to patients with thyroid cancer subjected to total thyroidectomy. Weight increments for both the surgical- or RAI- induced hypothyroidism groups were 10 vs 4 kg in the ATD or hemithyroidectomy-treated euthyroid groups vs only 0.6 kg in the surgically treated thyroid cancer cohort (78). Hence, these results suggested that thyroid autoimmune disease is associated with weight gain *per se.* The mechanisms underlying the direct effect of Graves’ disease on weight increase is still unclear. One hypothesis is that brown adipose tissue (BAT) expresses TSH receptors which are activated by anti-TSH-R stimulating antibodies of Graves’ patients. In fact, TSH may stimulate thermogenesis by binding to the TSH receptor expressed in adipocytes, a function involved in maintaining thermal status during hypothyroidism. Moreover, *in vivo* acute administration of recombinant human TSH at supraphysiological doses induced the release of small but significant amounts of leptin which was proportional to the adipose mass. Hence, it is reasonable to suppose that TSH stimulating activity may have per se an important effect on adipogenesis, mainly on white adipose tissue. On the other hand, this effect may be enhanced by inflammatory cytokines present in Graves’ patients (IL-1, IL-6 and TNFα) which are able to activate the hypothalamic, pituitary, adrenal axis, and cortisol secretion. In conclusion, several lines of evidence suggest that Graves’ disease is associated with excessive weight regain during therapy and may represent an important risk factor for the development of obesity. It is also possible, that the different effect of AITs on residual overweight may be due to their immunosuppressive effect (along with the use of corticocosteroids).

### Key points

Excessive weight regain may occur after treatment of hyperthyroidism, especially after radioiodine or surgery. Thyroid autoimmunity should be considered an independent mechanism that leads to inappropriate body weight increase.

## Therapy of hypothyroidism in obese patients: role of inflammasome, ubiquitination, glucurono- coniugation and activity of deiodinases

Replacement therapy with Levothyroxine (LT4) is currently the standard treatment for hypothyroid patients. Its therapeutic goal is a TSH value within the age-related range, which is associated with an improvement in symptoms, quality of life and cardiovascular risk. However, the most appropriate treatment of hypothyroid patients is still under debate, especially in some particular conditions including old-age and obesity. In particular, daily calorie intake and nutritional status may affect leptin levels, which, in turn, may upregulate hypothalamic TRH and deiodinase 1 (D1) activity (38, 41, 89–91). Therefore, obese patients show a higher average TSH level than non-obese subjects (61). This positive correlation between BMI and TSH may reflect a compensatory pituitary-thyroid axis activation by leptin and insulin (48) to increase energy expenditure. However, it may also suggest a certain degree of thyroid hormone resistance in obese patients (92) related to the chronic low-grade inflammation and inflammasome activation but also to the regulation of 5’ adenosine monophosphate-activated protein kinase (AMPK), a central target not only for the modulation of insulin sensitivity but also for the effect of thyroid hormones on appetite and energy expenditure (93). Indeed, *in vivo* studies performed in obese mice indicate that obesity-induced inflammasome activation up-regulates deiodinase activity by down-regulating ubiquitin-mediated protein degradation, thereby contributing to thyroid hormone (TH) resistance at pituitary level and body mass gain (94, 95). Moreover, impaired transport of TH through cell membrane by low-grade inflammation may represent an additional mechanism of thyroid hormone resistance in obese patients (96). Pituitary resistance and impaired cell membrane transport of thyroid hormones may explain the positive correlation between FT3 levels and body weight. Taken together, these studies suggest that TSH levels in obese hypothyroid patients may not recapitulate the adequacy of L-T4 replacement dose (61). Indeed, some studies suggest that optimization of L-T4 replacement dose should be performed according to lean body mass (LBM), rather than whole body weight (RBW). In particular, LBM is related to body mass cells (BMC) that corresponds to lean mass of body organs (liver, kidney and heart) and correlates to D1 activity, L-T4 glucurono-conjugation and basal metabolic rate (BMR). In contrast, fat mass, which is one of the major determinants of low-grade inflammation, may exert a confounding effect on L-T4 requirement (97, 98). Hence, further studies are needed to evaluate the role of different LT-4 dose and formulations or L-T4/L-T3 combination in the treatment of hypothyroidism in obese patients.

### Key points

The most appropriate treatment of hypothyroidism in obese patients is still debated. These patients may have some degree of resistance to thyroid hormone related to chronic low-grade inflammation and activation of the inflammasome.

## Bariatric surgery: impact on thyroid inflammasome, autoimmunity and thyroid function

Bariatric surgery has become a safe and efficacious treatment for patients with severe and complex obesity, who do not respond adequately to caloric restriction (99). Several studies reported a significant effect of bariatric surgery on many inflammatory markers, including thyroid autoantibodies, and a significant effect on thyroid hormone levels (100, 101). As discussed above, obesity is a condition associated with a low-grade systemic inflammation, leading to altered levels of various inflammatory markers (102, 103) and imbalanced adipokine secretion (104). For instance, adiponectin and orexin-A, two adipokines related to insulin sensitivity and calorie intake, are markedly decreased in obese patients along with a significant increase in inflammatory cytokines (105). Interestingly, there is evidence that patients with morbid obesity, subjected to bariatric surgery, display reduced levels of pro-inflammatory cytokines along with increased adiponectin levels that play an anti-inflammatory action (106). The effect of bariatric surgery in reverting the dysregulation of the inflammasome system has been extensively studied by Vicente et al. (107), who evaluated changes in inflammasome components in 22 patients with morbid-obesity before and after six months of bariatric-surgery (sleeve-gastrectomy and roux-en-Y gastric bypass). In this study, the most important changes were found in NOD-like-receptors, cell-cycle and DNA-damage regulators, while, at baseline, several components of the inflammasome were significantly related to metabolic alterations, including T2DM (CCL2/CXCR1/SIRT1), hypertension (AIM2/ASC/P2RX7) and dyslipidemia (CXCL3/NLRP7). It was interesting to note that the levels of all these markers significantly changed six-months after bariatric-surgery. Gene-expression levels of NLRC4/NLRP12/CXCL3/CCL8/TLR4 were related to pre and post-operative peripheral-blood mononuclear-cells. *In vitro* experiments, performed to validate the mechanistic insights of these findings indicated that NLRC4/NLRP12 silencing in the hepatoma cell line HEPG2 resulted in increased cell-viability and lipid-accumulation along with a reduction in the apoptosis rate. In respect to the relationship between inflammasome activation in morbid obesity subclinical hypothyroidism (108), a report by Zhu et al. indicates that obese patients subjected to laparoscopic sleeve gastrectomy display a significant decrease in inflammation markers, such as IL-6, TNF-α, and CRP (109) that is concomitant with a significant TSH reduction. In agreement with these results, some reports indicate a reduction/normalization in TSH levels in obese patients after weight loss induced by either bariatric surgery or a low-calorie diet (110). A recent meta-analysis confirmed that bariatric surgery is able to revert subclinical hypothyroidism in obese patients and to reduce L-T4 replacement dose. It was interesting to note that, while TSH, FT3 and T3 levels decreased significantly after bariatric surgery, FT4, T4 and rT3 levels remained unchanged (100). Although the association between bariatric surgery and thyroid function seems clear and mainly based upon thyroid autoimmunity, the mechanism of this relationship may be complex and bidirectional: some studies indicate a role for autoimmune subclinical hypothyroidism in the pathogenesis of obesity (111),, while others support the hypothesis that the excess of dysfunctional adipose tissue may be responsible for changes in serum thyroid hormone levels (90, 112). In line with this notion, Xia et al. reported a significant reduction in serum TPOAbs and thyroglobulin antibodies (TgAbs) in 101 patients with morbid obesity (BMI ≥ 32 kg/m2 or BMI ≥ 27.5 kg/m2 with one or more comorbidities) treated with bariatric surgery (101). The reduction in TPOAbs and TgAbs was from 79.3 and 177.1 IU/mL to 57.8 and 66.0 IU/mL, respectively (P < 0.05). In accordance with these results, Kyrou et al, albeit in a small population study, evaluated ultrasound thyroid morphology in obese patients treated with bariatric surgery and found a significant relationship between weight loss and an increase in thyroid echogenic pattern (113). In conclusion, these data indicate that bariatric surgery is able to attenuate inflammasome activity and may exert a beneficial effect on thyroid autoimmunity and function.

### Key points

Bariatric surgery can attenuate the activity of the inflammasome and can exert a beneficial effect on autoimmunity and thyroid function.

## Obesity, inflammasome and thyroid cancer

A functional relationship between chronic inflammation and cancer was first proposed by Virchow in 1863 and is supported by clinical and epidemiological evidence. As mentioned above, obesity is characterized by chronic low-grade chronic inflammation and insulin resistance. It is now widely accepted that the consequent deranged immunity, compensatory hyperinsulinemia, hyperlipidemia, and enhanced oxidative stress are risk factors for cancer development and/or progression (114–116). Several data indicate that increased inflammasome activity in adipose tissue is an important mediator of obesity-induced inflammation and insulin resistance. Inflammasome-induced cytokines are mainly produced from the hematopoietic compartment and act in autocrine and paracrine manners to form an inflammatory microenvironment (117), recruit immune cells and interfere with insulin signaling processes thus promoting the development of diabetes (118). Obesity increases oxidative stress that in turn upregulates inflammatory cytokines (119). Indeed, chronic inflammation activates transcription of factors such as nuclear factor kappa-light-chain-enhancer of activated B (NF-kB), STAT3, and activator protein 1 (AP1) in pre-malignant cells. All these pathways enhance cell proliferation and survival promoting angiogenesis in conjunction with hypoxia (120). Various infiltrating cells have been identified in tumors, namely, tumor-associated lymphocytes, tumor-associated macrophages (TAM), immature dendritic cells, mast cells and myeloid-derived suppressor cells. The presence of specific inflammatory-immune cells such as macrophages and mast cells in tumor sites has been associated with a poor prognosis of thyroid cancer (121). Thyroid cancer cells and the tumoral stroma may secrete several cytokines and chemokines, thus sustaining the survival of cancer cells promoting the selection of clones that acquire additional genetic lesions becoming resistant to oncogene-induced apoptosis. Proinflammatory cytokines produced in tumor sites by inflammatory and epithelial cancer cells depend mainly on the NF-kB transcription factor and are pivotal in cancer progression (121). Consistently, several cancer histotypes are more common and more aggressive in obese patients than in the normal weight population. Several lines of evidence suggest that obesity is also associated with thyroid cancer. Various epidemiologic studies and metanalysis have found a statistically significant association between BMI and thyroid cancer, especially in males (122). The risk of thyroid cancer appears to be positively related with the degree of obesity (123). Moreover, obesity is related to more aggressive histotypes (123). Animal models support these studies and explain the complex mechanisms between obesity and thyroid tumors demonstrating that a high-fat diet (HFD) induces thyroid cancer cell proliferation by enhancing cyclin D1 protein levels as well as the phosphorylation of RB protein, serum leptin levels and STAT3 expression (124). In addition, an inhibitor of STAT3 was able to inhibit the proliferation of HFD-induced thyroid cancer cells through the reduction of cyclins D1 and B1, CDK4, CDK6 and pRB (125). A role of inflammation and immune response in the onset of thyroid cancer and autoimmune thyroid diseases (such as Hashimoto’s thyroiditis and Graves’ disease) has already been demonstrated (126, 127). Moreover, increased TAM infiltration in poorly differentiated thyroid cancers (PDTCs) was found to be positively correlated with capsule invasion, extrathyroidal extension and poor prognosis (128). It has been also reported that the expression of BRAF V600E mutation and the RET/PTC gene rearrangement promote the activity of NF-kB, the expression of inflammatory mediators, and lymph node metastases in patients with papillary thyroid cancer (PTC) (129). Inflammation is intimately linked to the metabolic reprogramming of cancer cells. Indeed, compared with healthy tissues or benign lesions thyroid cancer is characterized by increased lactic acid production, regardless of its histotype. Moreover, PKM2, a key enzyme of glycolysis, has been found overexpressed in advanced and poorly differentiated thyroid cancer and associated with tumor aggressiveness and negative prognosis of PTC (130). In other cell models it has been shown that PKM2-dependent glycolysis promotes NLP3 inflammasome activation (131). Indeed, pharmacological inhibition of PKM2 attenuates the release of IL-1b, IL-18 and HMGB1 due to NLP3 activation (131). Although a direct link between thyroid cancer and obesity-induced inflammasome activation has not been clearly demonstrated, several lines of evidence support the hypothesis that a relationship may be also present in thyroid cancer. Recent studies indicate that the inflammasomes can be activated by fatty acids and high glucose levels linking metabolic danger signals to the activation of inflammation and cancer development. These data suggest that activation of the inflammasomes may represent a crucial step in the obesity-associated thyroid cancer development.

### Key point

Inflammasomes can be activated by fatty acids and high glucose levels linking metabolic danger signals to the activation of inflammation and cancer development.

## A possible role of vitamin D in autoimmune thyroid diseases and obesity

Several studies have shown that the risk and the clinical evolution of autoimmune thyroid disease and other chronic inflammatory diseases are associated with low plasma levels of vitamin D (132). This is not surprising as vitamin D modulates the synthesis of IL-1, IL-6, and other cytokines, suppresses dendritic cell differentiation, and influences regulatory T cell activity. Vitamin D inhibits the activity of TLRs (Toll-like receptors) by inhibiting the identification of pathogenic or non-pathogenic molecular patterns (e.g. LDLc binding with TLR in thyroid cells) by TLRs. In addition, vitamin D *via* its receptor (VDR), inhibits the deubiquitination and activation of NLRP3 inflammasome (133, 134). Notably, hyperthyroidism is associated with reduced levels of vitamin D (135). Moreover, vitamin D negatively modulate lipogenesis controlling calcium influx into adipocytes (136). These data are in agreement with studies showing that vitamin D deficiency is associated with overweight, obesity and insulin resistance (137, 138). Accordingly, vitamin D supplementation may decrease the incident risk of autoimmune disease (139) and increases insulin sensitivity (140, 141).

### Key points

Vitamin D attenuates the activation of the inflammasome and its immune response, modulates lipogenesis and increases insulin sensitivity. Vitamin D deficiency is related to the risk and severity of thyroid autoimmunity and obesity.

## Conclusions and perspectives

Recent epidemiological and experimental data provided evidence of a bidirectional interaction between obesity and thyroid autoimmunity. In particular, obesity is associated with an enhanced risk of hypothyroidism and of developing anti-thyroperoxidase antibodies. This is also true for thyroid autoimmunity related disorders like GO: low-grade inflammation and inflammasome activation in dysmetabolic subjects may enhance the autoimmune process underlying GO pathogenesis and its clinical and phenotypic expression. On the other hand, thyroid autoimmunity may favor overweight and obesity. One empirical evidence supporting this hypothesis is Graves’ disease. Indeed, a marked weight gain during GD therapy may represent an important risk factor for the development of obesity. Moreover, the lower extent of weight gain with immunosuppressive therapies unravels the possibility that thyroid autoimmunity *per se* is able to favor obesity, although the exact mechanisms underlying this phenomenon are not fully understood. In addition to the positive effect of obesity in thyroid autoimmunity and hypothyroidism, several lines of evidence suggest that obesity related inflammasome activation may induce a certain degree of TH resistance both in peripheral tissues and hypothalamus leading to a significant increase of TSH, thereby exerting confounding effects on L-T4 requirement in obese people. Inflammasome activation in obesity may also increase the risk of thyroid cancer and is related with a more aggressive thyroid cancer phenotype. Several *in vitro* data support and explain this observation. A further observation confirms the role of inflammasome related obesity in thyroid disorders: bariatric surgery is able to attenuate inflammasome activity and may exert a beneficial effect on thyroid autoimmunity and function. Taken together these results indicate that thyroid function should be always screened in obese people and that thyroid disorders should be carefully addressed in obese and dysmetabolic people. Further studies are needed to unravel the mechanisms underlying the bidirectional relationship between obesity and thyroid disorders as well as to evaluate the role of different LT-4 doses and formulations or L-T4/L-T3 combination in the treatment of hypothyroidism in obese patients.

## Ethics statement

Written informed consent for participation was not required for this study in accordance with the national legislation and the institutional requirements. 

## Author contributions

Conceptualization, RM, AB, VV, FF; methodology, RM, VV; software, RM, AN, TP; validation, AB, FF, RM,VV; investigation, RM, TP, VV, FF, AB; resources, RM, FF, AB; writing—original draft preparation, RM, FF, AB, VV; writing—review and editing, RM, FF, AB, VV; supervision, RM, FF, AB, VV; project administration, AB. funding acquisition, AB. All authors have read and agreed to the published version of the manuscript.

## Funding

This research was supported in part by Fondazione AIRC: IG n. 23369 to AB and by Ministero della Salute, Italy, grant RF-2019-12368937 to AB.

## Conflict of interest

The authors declare that the research was conducted in the absence of any commercial or financial relationships that could be construed as a potential conflict of interest.

## Publisher’s note

All claims expressed in this article are solely those of the authors and do not necessarily represent those of their affiliated organizations, or those of the publisher, the editors and the reviewers. Any product that may be evaluated in this article, or claim that may be made by its manufacturer, is not guaranteed or endorsed by the publisher.
